# "The Hospital" Nursing Mirror

**Published:** 1899-09-30

**Authors:** 


### "The Hospital" Nursing Mirror.

Being the Nursing Section of "The Hospital."

[Contributions for this Section of "The Hospital" should be addressed to the Editor, The Hospital, 28 & 29, Southampton Street, Strand, London, W.C., and should have the word "Nursing" plainly written in left-hand top corner of the envelope.]

### Notes on News from the Nursing World.

### NURSES FOR SOUTH AFRICA.

The following army nurses have proceeded to South Africa: Superintendent Miss M. Russell, and Nursing Sisters Miss A. Sammont, Miss E. Martin, Miss J Hoadley, and Miss M. G. Hill. We are authorised to add that it is not contemplated by the War Office to employ civilian nurses.

### THE NURSING STAFF AT UNIVERSITY COLLEGE HOSPITAL.

The following paragraph appeared in a prominent position in the *Daily News* on Monday:—

"For the last forty years the Sisters of All Saints' Church, Margaret Street, Cavendish Square, have been closely connected with University College Hospital, and a staff of thirty-two sisters, under a chief sister, have done nearly all the nursing, and the chief sister has had supreme control over all the nursing and domestic arrangements, and the ordinary nurses, wards-maids, and servants have looked to her for guidance. In return for all their services the sisters accepted a merely nominal remuneration. The committee of the hospital, having taken the supreme control out of the hands of the chief sister, and vested it in the hands of others, all the sisters have, with her, withdrawn for good from the hospital."

In reference to this announcement it may be stated that it is now a month ago since the thirteen Sisters of All Saints', Margaret Street, and some of the nurses retired from University College Hospital; that on September 1st the new matron entered upon her duties at the hospital; and that from the time of her installation she has had the cordial co-operation of a full staff of nurses.

### A PERMANENT NURSING HOME FOR LLANDUDNO.

At the annual meeting of the Llandudno District Nursing Association Lord Mostyn said "he hoped to live to see the day when a permanent nursing home would be established in the town." Such an expression of opinion from a peer who is in a position to materially assist in the fulfilment of his hope should give a great impetus to the movement. Llandudno, as one of the largest watering-places in the Principality, would be a very appropriate centre for a nursing home, especially as, owing to its fine air, the numerous visitors include many invalids. In the year which has just expired, the single nurse paid more than 4,000 visits, a fact which confirms the view that more nursing help is already wanted.

### THE IMPROVEMENTS AT KING'S COLLEGE HOSPITAL.

Having enjoyed a holiday of nine weeks, the nursing staff at King's College Hospital have now returned to duty. The matron alone has not been away from her post all the time the hospital has been in the hands of the workmen engaged in the task of relaying the floors and of installing the electric light. It was not an easy matter to distribute the patients among other institutions, but the improvement effected in the appearance of the wards and the additional comfort, quite justifies the expenditure of time, trouble, and money. Nothing has been done to the quarters of the nursing staff, because they were admirably arranged long ago. The medical staff reassembles with the opening of the winter session of the medical schools, and in November the regular nursing lectures begin. Probationers are accepted all the year round, and they are drafted into the wards every month.

### A LOSS TO THE NURSING WORLD.

By the sudden death of Surgeon-General Lithgow, medical superintendent of the Royal Infirmary, Edinburgh, from pneumonia supervening on a chill, the nurses of the Edinburgh Infirmary in particular, and the nursing world in general, have sustained a severe loss. "Every army sister, and every nurse," writes a correspondent from Edinburgh, "who has served under him, will feel his death; and the lady superintendent especially, as she not only mourns a valued friend, but will need a new colleague in the management of the infirmary and the medical nursing school, of which the Scottish people are so proud. All the nurses concur in testifying to the great interest the Surgeon-General took in improving the accommodation for the staff. The nurses' home is one of the most perfect I have ever seen, and I have had the privilege of going through all the nurses' homes of the London hospitals. It is to the late medical superintendent and the present lady superintendent that the infirmary nurses are indebted for the comfort and convenience of their splendid quarters."

### THE "HUMOUR" OF POOR LAW GUARDIANS.

Various reasons are assigned for the dearth of applicants for nurses' posts at workhouses. Perhaps the subjoined report from the *Eastern Evening News* of September 22nd may throw a little fresh light upon the subject:—

"At the meeting of the West Ham Guardians yesterday, the appointment of a night nurse occupied attention. One of the applicants had a rather red face with a few spots on it, which she said was occasioned by a visit to the seaside.

"The Rev. Tom Warren: A friend of mine who looks like that says it is through eating pork. (Laughter.)

"Mr. Anderson: Well, it's not a nice thing for a patient to see.

"Mr. Skinner: They want cheerful faces.

"Rev. Tom Warren: Then I shall move that some members be not allowed to go inside the infirmary. (Roars of laughter.)

"Mr. Judge: I think we had better advertise in future, 'Only people with cheerful faces need apply.' (Laughter.) My experience is that the plainest women are the kindest.

"Rev. Tom Warren: Appoint the woman, she'll be all right.

"The applicant was appointed."

It is not surprising if nurses generally shrink from being subjected to such "pleasantries" as those of the West Ham Guardians.

### SEATS FOR NURSES.

A serious, but rather vague, complaint is made in the columns of one of the Dublin dailies in reference to the accommodation provided, or not provided, for nurses. It is stated that in "many of the hospitals" the nurses who are serving their time "are obliged to attend from seven a.m. until nine p.m. with only a short interval for meals," and that "they are not allowed to sit, nor is seating accommodation provided." If this is so, the most requisite alteration is in the hours, which are much too long. But that they should be maintained without affording the nurses the opportunity of sitting down when they are not attending on the patients is almost incredible. The complaint is made at the instance of a lady, whose daughter has been in one of the hospitals; but in such cases reform is not likely to be accomplished without naming the offending institutions.

### THE SAD INCIDENT AT KENSINGTON INFIRMARY.

A most unfortunate occurrence took place last week at the St. Mary Abbot's Infirmary. A boy, a little over two years of age, fell out of bed, and in the temporary absence of the nurse, was suffocated by the pressure of the band of his bed-jacket on the throat. It appears that he was in a small ward with two other children, as he was exceedingly restless, and showed symptoms of sickening for measles. When the nurse in charge had to leave the ward to fetch the dinners, she tied the child in bed by passing a length of bandage through the two top buttonholes of his bed jacket, and fastening it to the head of the bed. We are informed that the neck-band of the jacket measured fourteen and the child's neck eight inches, so that it was very loose. In spite of this precaution, he managed to climb out of bed, and slipping, he was suspended by his jacket to the bedstead. Unfortunately, his weight fell on the throat, and when the nurse returned he was dead. At the subsequent inquest the jury returned a verdict of "Death from Misadventure." In consequence of this lamentable occurrence, the authorities at Kensington Infirmary have given orders that restless children are not in future to be tied in bed.

### "CRAMPING, BUT QUITE SUITABLE."

The Hereford Board of Guardians have appointed a superintendent nurse at the workhouse. It was intended to open a lying-in ward and to provide better accommodation for nurses before taking this step; but after the chairman and vice-chairman had inspected the house with the master and matron, they came to the conclusion that the existing accommodation, "though cramping, was quite suitable." This curious decision being, apparently, endorsed by the Board, they were able to unanimously agree on the appointment of Miss E. Evans as superintendent nurse, and to decide that the nurses should wear a cotton uniform. We can only hope that the nurses will not find the cramping accommodation so unpleasant that, in spite of its suitability, they will be obliged to migrate to less restricted quarters.

### NURSES' BICYCLES.

It is a remarkable sign of the times that when the plans for the new Richmond Infirmary were under discussion, it was pointed out to the architect that he had made "no provision for the nurses' bicycles." The architect confessed that he had not taken the "bikes" into account, but added that he could easily remedy the deficiency if required. On future occasions, since bicycles have become so largely a necessity for nurses, it would be worth while in extensive building operations to include a small outhouse for the stabling of the machines.

### THE RESIGNATION OF THE MATRON OF HANGLETON HOSPITAL.

General regret appears to be entertained, especially among the poorer inhabitants of Hove, at the resignation of the popular and capable matron of Hangleton Hospital. According to a local newspaper, the matron, Miss Whitaker, met with an accident the other day, falling down some stairs and seriously hurting herself. She was not able, in consequence, to perform her duties of supervision as thoroughly as usual, and omitted to notice a small neglect on the part of a member of the staff. This neglect was, it is stated, discovered by the chairman of the Sanitary Committee of the Town Council, "who personally reprimanded the matron in such a way that there was no other dignified course left her than to resign." It is admitted, however, that the matron did not report herself off duty. We agree with our local contemporary that the matter is one which ought to receive full investigation.

### NURSING IN BOMBAY.

The three European lady nurses at the Sassoon General Hospital, Poona, are all down with enteric fever. Miss McCartney is now almost convalescent, but Miss Sladen and Miss Slater are still in a critical condition.

### FROM PALESTINE.

The Edinburgh Medical Missionary Society is doing good work in Damascus. The hospital opened there last summer is called the "Victoria," and it is gratifying to learn that one large and one small ward have been entirely fitted, furnished, and decorated by the Diamond Jubilee Fund, subscribed for by the British residents in Syria and Palestine. The patients at this hospital represent all classes, both from Damascus and from the country round; but, as is truly said, "only the bare fringe of the work can be touched." A hospital of 40 beds, and a population of about 300,000! "What," indeed, "are these among so many"?

### SHORT ITEMS.

A number of Sisters of the Indian Army Nursing Service have volunteered for service in the Transvaal in the event of hostilities. It is understood that at least four ladies will join the Indian contingent. They will accompany the three Indian field hospitals warned for service, but will, of course, be transferred to the base hospital at Pietermaritzburg on their arrival in Natal. The Moreton Hampstead Nursing Association is to be congratulated upon the provision of an endowment, by Mr. George Wills, for a nurse, and the gift, by the Hon. W. F. D. Smith, M.P., of a sum for the erection of a cottage hospital.—The Secretary for Scotland has signified his approval of a contribution by the County Council of Lanarkshire of £50 out of the Equivalent Grant for the purpose of assisting in the training of nurses by the Scottish branch of the Queen Victoria Jubilee Institute for Nurses as a scheme of public utility, in terms of the Scottish Education and Local Taxation Account Act, 1892.—A jumble sale and sale of work in the grounds of Harston Mills resulted in an addition of £15 to the district nursing fund. The villages comprised in the district are Little Shelford, Hauxton, Harston, and Newton.

### The Nursing of Cases of Hernia.

By W. McAdam Eccles, M.S.Lond., F.R.C.S.Eng., Assistant Surgeon West London Hospital.

II.

In the preceding lecture the preparation of a patient for an operation upon a strangulated hernia was dealt with. It is now our purpose to look into the duties of the nurse during the actual operation itself. She should give her attention then chiefly to the operator, and should as far as possible anticipate his every want, in the way of swabs for sponges, lotions for his hands and the patient's skin. It is essential that the nurse should carefully cleanse her own hands before attempting to take any active part in assisting at the operation, and she should be on her guard against fouling them again after they have been washed. She should prepare and arrange the requisite dressing after reference to the wishes of the surgeon in the matter, and she should see that the bed is ready to receive the patient at the end of the operative procedure. When the operation has been completed it will be her duty to assist in replacing the patient in bed, in the most comfortable and convenient position. It is usual to make the patient assume the dorsal posture with a pillow placed under his knees, so that they may remain passively flexed over it. Prompt attention should be given to any efforts on the part of the patient to vomit, and it may be well that the region of the wound should receive some outward support by the pressure of the nurse's hand if the straining produced by the emesis is severe.

Nothing but a few sips of hot water should be given by the mouth for some hours after the operation. The nurse will do well to find out what is the individual surgeon's line of treatment with regard to food subsequent to the operation of herniotomy. Personally I consider that in many cases the patient, who has been considerably depressed by the repeated vomiting, requires food chiefly as a stimulant as soon after the operation as is consistent with his attempts to vomit. A little hot milk or hot beef tea often has the effect of stimulating an otherwise fainting patient in a remarkable manner. Absolute quiet is essential, and friends should be rigidly excluded from the patient's presence until allowed by the doctor.

After operations for herniæ there is not infrequently some amount of difficulty on the part of the patient in passing his urine. This arises to some extent from the position of the sufferer as he lies on his back, but it is in part the outcome of reflex action. The surgeon should always be informed of the occurrence of this difficulty, for it may be necessary for a catheter to be passed. It is advisable, if possible, to avoid any narcotic, although the patient may complain of some amount of pain. It will exert all the ingenuity of the nurse in many cases to promote the comfort of the patient during the first night or two after the operation. The patient finds it very trying to continue in the dorsal position, and it will need many alterations in the adjustment of pillows by the nurse to maintain any degree of ease. One fact it is very important for the nurse to notice, and that is whether the patient passes any flatus after the operation, and also whether any fæcal material finds its way out through the anus. In a few cases, in a surprisingly short space of time after the patient has had his gut liberated, the intestinal contents will reach the end of the bowel, but in others again it may be a good many hours before even flatus escapes. If either flatus or fæces make their appearance it is to be considered a satisfactory sign, since it proves that the intestinal obstruction which was present prior to the operation has been entirely relieved by it.

Vomiting, if it persists for more than two or three hours after the operation, is a bad sign, and it may indicate that the bowel has not been entirely freed from the constricting force, or that it has been so much damaged that it is unable to recover. Continuance of emesis should be promptly reported to the surgeon. Sometimes this after-vomiting is due to the effect of the anæsthetic, and it will then often yield to the administration of a drachm of brandy in a teaspoonful of hot water, or to two or three drops of tincture of iodine. After all vomiting has ceased, and there are no longer any signs of intestinal obstruction, and if the surgeon approves, the patient may commence taking solid food, in the form of bread and milk, custard, eggs, and, ere long, fish and minced meat. · He may resume his ordinary diet at the end of a week or ten days in most cases. The nurse must on no account administer a purgative to the patient on her own initiative. It is usual to allow the bowels to act spontaneously, but if such an action does not occur by the morning of the fifth day the surgeon will probably order an enema. Often a glycerine or small oil injection is sufficient, for it is desirable to disturb the bowel as little as possible after an operation for the relief of strangulation. Generally, when the bowels have acted the patient may be considered to have passed the critical stage in the period of convalescence.

The temperature of the patient has to be carefully recorded, and any rise may indicate that there is some septic process taking place in the wound, and this may call for the dressings to be changed. If a drainage tube has been inserted it is usual to remove it at the end of thirty-six or forty-eight hours, so that the nurse should have a fresh supply of dressings ready for this. If there is no indication of any untoward changes in the wound, the dressings are left undisturbed until the eighth, ninth, or even the tenth day, when they will be removed and the sutures cut out. The length of time that a patient is to be confined to bed after herniotomy varies in the practice of different surgeons, but the patient may in most instances be allowed on a couch at the end of a fortnight, and soon after to sit up, while at the end of the third week walking may be commenced. It is, perhaps, not advisable to permit the patient to exert himself too much by moving about unless he has been provided with a truss to support the hernial region.

The other class of case in which an operation is undertaken for hernia is one in which there is no suspicion of any strangulation, but the protrusion is dealt with by operation so that an attempt may be made to completely rid the sufferer of it. In such a proceeding the operation is not one of emergency, and sufficient time can be allowed for a more thorough preparation. It is usually my custom to prepare, or instruct a nurse to prepare, the area which will be the site of the operation two days before this is undertaken. Supposing that the operation is to be performed at nine a.m. on a Wednesday, on the previous Monday morning the patient should take a hot bath, paying particular attention to the cleansing of the hernial region. Directly after the bath the parts should be shaved, and again scrubbed with soap and water, and the soapsuds removed with recently-boiled water. The skin of the operation area is now to be washed over with ether or turpentine, and then with a 1 in 1,000 solution of the biniodide of mercury, after which a dressing of the double cyanide of mercury and zinc gauze is to be applied, and so fixed that it cannot shift. The whole process is to be repeated on the Tuesday morning, at which time also the patient should be given a smart purge, so that the bowels may be thoroughly evacuated during the day, and the patient thus not disturbed in the night preceding the operation. On the Wednesday morning no food should be taken prior to the operation. An enema should be administered at half-past seven a.m. On the operating-table the surgeon will again cleanse the parts, which should thus be rendered quite aseptic. The after-treatment of a case of radical operation upon a hernia is much the same as that which has been already described in the operation for the relief of strangulation. There is not, however, the same amount of anxiety, since the intestine is in its normal condition, and the patient probably in a state that is satisfactory for the success of an operation. Care should be taken that the wound is supported if there is much vomiting from the effects of the anæsthetic. The bowels can usually be allowed to act of their own accord, and there need be little or no restriction in the diet of the patient after the first day or two. The period of convalescence should not be any longer than that of a patient after herniotomy for strangulation.

### Consumptives' Mode of Living in Davos.

By a Sister in the Provinces.

It may be of interest to some of the readers of the "Nursing Mirror" to know a little about Davos Platz and how the poor consumptives pass their time away, as well as some of the treatment. Davos Platz is a delightful health resort, situated in Eastern Switzerland, and stands over 5,000 feet above the sea level. The average breadth of the valley is about half a mile, and it is protected on both sides by vast mountain ranges, which rise for the most part precipitously to between 2,000 and 5,000 feet above the valley—consequently, between 10,000 and 11,000 feet above sea level. The position of Davos—lying N.N.E.-S.S.W.—insures it many hours of sunshine on the briefest winter day. The soil is dry and gravelly, and there are numerous level walks. The air is very strong, and possesses tonic properties to a high degree. The patients begin to assemble in Davos from September to November, when it is usually pretty full; they reside either in hotels or sanatoriums. When they first arrive, more especially the bad cases, they find the air rather strong and tiring. Accordingly for the first fortnight they usually take life very quietly. Until November, Davos is supposed to be "asleep." During the "sleepy" time the patients do little besides sitting out on their balconies or taking short walks. When patients who have been to Davos before first arrive, they either call on their medical man, or send word that they have arrived; then he visits them or they visit him, as the case may be. Soon after their arrival they are asked to send a sample of their expectoration to the analyst, and he forwards a report to the doctor; it is then compared with the last winter's report. I have known of cases in which patients have asked the analyst for a copy to gratify their own curiosity. Their treatment is really very little more than the pure fresh air. A good many of them do not receive regular visits from their doctor unless the need arises. They are out of doors the greater part of the day. In the evening after dinner many also go out warmly tucked up on couches with fur rugs and hot bottles. They call this "doing the cure." It is rather difficult to know how to dress, as the mornings till about half-past ten are bitterly cold, so that really one cannot be too warmly clad; but by half-past eleven the sun is in full power, and one wants cotton blouses. The sun shines till about half-past three p.m. The sunset tinting the most lovely mountains is magnificent. By four o'clock most people are in their furs once more, so that in Davos one has summer and winter almost every day. There are, of course, some days when there is hardly any sun at all, but these are very rare. The autumn colouring of the mountain ash is superb.

As to the treatment of the patients residing in hotels, some find it far preferable to a sanatorium, which so often gives one the idea of a hospital. To the nervous and depressed the hotel is more like home life than the sanatorium, although in the latter the patients have all that could be desired with regard to comfort, and all that is good for them. But the question after all is, Do not the vast majority of consumptives require supervision? Surely, fresh air cannot suffice to cure the disease. Take, for instance, the case of a young lady who went out alone last year. She was usually of a bright and intelligent disposition, but she felt her illness more than anyone knew. She had fits of depression, and would take her food or leave it just as she felt inclined. Sometimes, she would sit out on her balcony long after the sun had gone done with no extra wraps, or she would walk too much one day and have to stay in bed the greater part of the next in consequence. This is not an unusual case, but I hope it may impress some who have members of their family about to winter abroad, and that they will well consider the matter. It cannot be a kindness to allow a person whose life depends on her common sense to do just as she likes with no one to advise her or check her folly.

Apart from this drawback, hotel life in Davos is all that could be wished for, and I believe most people in the hotels have a very happy winter home. The daily routine is as follows: Breakfast is from eight till ten—the time for the first meal is always optional, and a great number of patients breakfast in bed, for which a little extra charge is made. Lunch is at one p.m. No tea is provided by the hotel, but I think most people have their spirit kettles and stoves in their own rooms, and make a cup of tea about four o'clock. Dinner is at seven p.m., and the food is very good and well varied. The band plays every day, and every hotel has it to itself twice a week. It plays outside on the balcony one morning a week, and in the hall after dinner till ten p.m. one evening, and it is greatly appreciated by most of the patients.

There is one sad feature in Davos. A good many of the patients are forbidden to enter a church on account of the atmosphere being impure and bad for them. The result is that most of these poor things—some of whom are spending the last years of their lives there—live on without entering a place of worship. There is one English church, and a chaplain who is very kind and attentive to all who desire his help. But it strikes one as being somewhat strange that the greater part of the patients are allowed to go to amusements for hours in places where I am sure the air is quite as impure as that in church, where service lasts only an hour.

### Minor Appointments.

Borough Fever Hospital, Ipswich.—Miss Edith Bangham has been appointed Charge Nurse. She was trained at the Royal Devon and Exeter Hospital, and remained there eight years, taking the duties of staff, sister, or private nurse, according to requirements.

Bethnal Green Infirmary.—Miss Florence Mary Scott Cavell has been appointed Superintendent of night nurses. She was trained at the Metropolitan Hospital, and has since been charge nurse at the Fountain Hospital, Lower Tooting, and ward sister at Poplar and Stepney Sick Asylum.

Oxford Workhouse.—Miss Mabel Reade has been appointed Assistant Nurse. She was trained by the Meath Workhouse Attendants' Association at St. Peter's Home, Kilburn.

Hertford British Hospital, Paris.—Miss Mookley has been appointed Staff Nurse. She was trained at West Ham Hospital, Stratford.

Totnes Union.—Miss Rebecca Jeans has been appointed Assistant Nurse. She was trained by the Meath Workhouse Attendants' Association at Blenheim House, Kew Gardens.

### Nursing Under the Metropolitan Asylums Board.

### CHAT WITH THE MATRON OF THE NEW FEVER HOSPITAL.

By Our Commissioner.

It was with two objects in view that I paid a visit to the newest of the fever hospitals erected under the auspices of the Metropolitan Asylums Board—namely, to personally inspect the nursing accommodation provided, and to obtain any information I could that would bear, in a practical manner, upon the question of training and the position of nurses in the employment of the authorities at Norfolk House. The Grove Fever Hospital at Lower Tooting is immediately opposite to the Fountain Hospital, and was only opened last month. As was the case when the Fountain Hospital was opened, Dr. Foord Caiger has been specially appointed as medical superintendent, and Miss Helen Wacher matron during the first six months.

Miss Wacher conducted me over the Nurses' Home, and I also went into one of the very large, lofty, and airy wards reserved for scarlet fever patients. Parenthetically, I may say that cases of scarlet fever, enteric fever, and diphtheria are isolated in separate blocks. The Nurses' Home possesses every possible comfort and convenience. The entrance hall is fitted up as a kind of small winter garden, and contains a piano. There are separate commodious and tastefully furnished sitting-rooms for charge nurses and assistant nurses, though by an ingenious arrangement they can be converted into one if necessary. But while the principle of dividing the grades is observed throughout, the accommodation and appointments, whether of the sitting-room or the dining-room, are equally good. The same remark applies to the bedrooms. There are separate blocks for day and night charge and assistant nurses, but each of the nurses has a dainty little bedroom to herself, and all are fitted up alike. Not only is each bedroom numbered, but so is every article in it, including the bedding and linen. There are bath-rooms, linen-rooms, and box-rooms. No detail that can make the nurses feel thoroughly at home seems to have been neglected, and I was, therefore, not surprised to learn that for months the work of furnishing, sewing, and making the linen and clothing has been going on.

Chatting subsequently with Miss Wacher, who has been matron of the Hospital Ships since 1884, I inquired the number of the staff of nurses at the present time.

"Sixty. But," continued the matron, "we are hardly in working order yet. The number will be between 160 and 170, and there is sleeping accommodation for more. The patients are increasing rapidly; there were 115 in the hospital last night."

"Just now the question of nursing at the fever hospitals is exciting particular attention. You have shown me how excellently the nurses are provided for in the Home here. Do you consider that they are liberally remunerated?"

"The charge nurses receive £36 a year on entering, and an increase of £1 a year up to £40; the assistants of the first class start with £24 and rise at the rate of £1 a year to £28; assistants of the second class start with £20 and rise at the same rate to £24. That is, of course, if they have satisfactorily discharged their duties. No salary is advanced except by the committee on the recommendation of the principal officers."

"What about off-duty?"

"Twelve hours off-duty weekly are allowed to each nurse, and one whole day every month. The eight hours are usually taken from two to ten, but the nurses have the privilege of going out from half-past eight to ten p.m. if they please. I think that the Board are very liberal in the matter of off-duty."

"And holidays?"

"The assistant nurses have three weeks' annual holiday, and the charge nurses a month. All the leave has to be taken at a time when it is convenient to the hospital."

"You have mentioned three classes of nurses. Are there any probationers?"

"None. The charge nurses have had three years' training in a hospital. No training is necessary for the second assistants, but the first assistants must have had at least one year's experience of nursing in a general hospital or poor law infirmary. The second assistants are eligible for promotion after two years to the rank of first assistants."

"Is it a fact that the charge nurses do not remain for more than a short period in fever hospitals?"

"I have often heard of them leaving in six months. They learn a good deal about fever nursing in that time, and I think that it is a great advantage to them to have the experience. Many, of course, remain longer."

"Do the assistant nurses stay for many years?"

"Yes, a good many of them remain under the Board for years. If a nurse makes an application to the medical superintendent and the matron to be transferred to another hospital to be nearer her friends, it is submitted to the committee for their sanction, and the transfer is often allowed."

On the question of trained and untrained nurses, the matron said:

"There are some very good nurses who have not received any general training, while on the other hand every trained nurse is not suitable for fever work. For a nurse to possess a knowledge of ward management is more important in these hospitals than in a general hospital, in which the wards are under the charge of a sister."

"It is said sometimes that the charge nurses find it difficult to obtain adequate help from the assistant nurses who have not been trained?"

"This to some extent, no doubt, is true, but the charge nurse has the remedy largely in her own hands. She has to manage her assistants, and she is expected to teach them as far as she can."

"Much as a sister has to manage her staff?"

"Yes, but there is an essential difference between a charge nurse and a sister. I was rather surprised to hear a charge nurse say that before she came to a fever hospital she thought the duties were the same as those of a sister. The work is not the same. Whereas a sister is always in charge of her ward, a charge nurse is only in charge during the day or the night, as the case may be."

"What hour does she go on duty in the day?"

"At seven. She is off duty at eight, and has no more to do with the ward until the next day."

"Then there is the disadvantage of being cut off on account of the infection from free social intercourse."

"Yes, that is inevitable. We are different in that respect from an infirmary, or a general hospital, and must be cut off. But every nurse takes up the work with the distinct understanding that she will have to put up with certain drawbacks."

"As, for example, night work."

"Quite so. The charge nurses have frequently an objection to night work. Still, the conditions of service under the Board are known to all who enter it before they are appointed, and my experience is that, as a rule, the nurses, whether they are charge or assistant nurses, are very well satisfied with the regulations and the manner in which they are treated."

Certainly, apart from questions of controversy, if all the hospitals belonging to the Asylums Board resemble the Grove, with its spacious corridors, its bright appearance, its up-to-date arrangements, including the electric light, and its ample provisions for the nursing staff, they need not fear comparison with other institutions.

### Echoes from the Outside World.

AN OPEN LETTER TO A HOSPITAL NURSE.

These are days in which we always appear to be demonstrating, and if we are not demonstrating we are celebrating. For instance, there was a demonstration in Trafalgar Square on Sunday, and there is to be another shortly, while we have barely ceased to demonstrate our sympathy with Captain Dreyfus in prison before we are invited to celebrate his release from prison by a banquet on Tuesday week. So long as some regard is paid to the susceptibilities of the French nation, and temporate language only is used, the latest celebration will, I suppose, do no harm, though one wonders whether it is really being got up to glorify Dreyfus or certain people in London. With regard to Trafalgar Square, I cannot help asking whether there is any need to demonstrate confidence in the action of the Government? So far as the man, or the woman, in the street is concerned, I should have fancied that the proceedings last Sunday sufficed. It is true that the advertised object of that demonstration was not exactly friendly to Ministers, but either the people who assembled to protest remained to praise, or the great majority of the company went with a very different motive from the organisers of the gathering; and if the crowd demonstrated anything, they demonstrated that their sympathies are with Lord Salisbury and his colleagues in the present crisis, as against those who seem to be of opinion that the interests of peace, and of England, would be safer in the hands of Mr. John Morley, Mr. Leonard Courtney, and Dr. Clark. There was no disorderly conduct to speak of on the occasion, and the police appear to have acquitted themselves admirably. But it is so easy to convert outdoor demonstrations into dangerous squabbles that you will probably agree with me that the fewer we have of them the better, whatever their avowed or actual object.

---

I assume that, so far as the outside public are concerned, the advocates of rational dress did their cause harm instead of good by the proceedings at their meeting on Saturday at Reading. It was a jubilee meeting, and about seventy members, headed by Lady Harberton, left Hyde Park soon after one o'clock, arriving at Reading about half-past six. This was pretty good riding, considering the strong head-wind which had to be faced. A good deal of chaff, more or less polite, was levelled at the riders as they made their way out of London, though Lady Harberton assured her hearers at the dinner that the movement was getting on well, and that after passing Hounslow they had met with no opposition save a little hooting from a few small children. But the expression of a desire to exclude the toast of Her Majesty because "the Queen doesn't like bloomers," and the statement of the President of the league that "the Almighty must have meant women to wear trousers, or he would have given her a tail in place of her legs" will not incline the most loyal and reverent of England's daughters to become "Bloomerites." I can quite believe that rational dress is much nicer and more convenient to ride in than the ordinary skirt, and that there is not the slightest need for it to be unbecoming. But if the wearing of "rational" dress, as it is called, brings in its train, as well as the imperative need of a cigarette, a contempt for the Sovereign and a flippant way of speaking of God's creation, I fancy a good many cyclists will continue—to quote Madame Sarah Grand—to have an "objection to expose the British matronly ankle." The author of "The Heavenly Twins" says that as long as this prejudice goes on "no good will come of it." Well, then, some of us must remain content to be condemned as creatures who stand in our own light and in that of other people.

---

According to the speeches of Sir Charles Cameron and Professor Smith at the Health Congress at Blackpool on Saturday, modern brandy is a very different product from the old French article, on which medical prescriptions were formerly based, and complaints are made that the results of taking the modern French brandy are frequently not such as the doctors expect. This, it is stated, is because, owing to the outbreak of phylloxera in French vines, brandy is now largely made from corn and potatoes, and even beetroot and molasses, so that the chemical properties differ considerably from those to be found when the stimulant is made from grapes. But as natural methods of distillation are still the rule in Spain, the Spanish product appears to be less open to the charge of artificial processes than the French, and when prescribed for medical purposes more likely to produce the desired effect. If so, most of us, and nurses especially, would be glad to use the Spanish, in preference to the French, cognac. But we have so long depended upon France to provide our supply that it is not easy, on the spur of the moment, to indicate where to purchase a good article of Spanish manufacture. I am told that, at present, Spanish brandy is mostly used for blending purposes only, and that the flavour of sherry about it would militate against its becoming a favourite with the British public.

---

I recently came across three cases in one morning's paper which serve as illustrations of the utter inability of the modern child to stand reproof of any sort, and of the entire absence of desire on the part of the parents that punishment should be inflicted upon the little offenders. On Saturday a little girl of thirteen was charged at Chesterfield with deliberately attempting to commit suicide in the canal at Staveley because her parents had accused her of stealing twopence. On the same day a boy of nine was reported missing. His father had chastised him for some childish misdoing, and he was believed, in consequence, to have run away from home with two older lads. The third case was that of a small girl at the Gill Street Board School, Limehouse, who had been put in the corner with her face to the wall for five minutes. One would hardly call such a mild punishment excessive, but that was evidently the opinion of the child's mother. Happening to come on the scene just after her daughter had been released from durance vile, she indignantly asked the child, "Why didn't you kick her and bruise her legs?" and finding that the governess had not been favoured with this sort of treatment, she proceeded to do what she could to remedy matters by shaking the instructress violently, abusing her roundly all the time. The fact that the magistrate made strong remarks on the woman's conduct and fined her well, will not, unfortunately, ensure her teaching her child to behave decently for the future.

---

Those of you who love a musical treat, when you can get an evening "off," will perhaps like to know what the Royal Choral Society proposes doing through the coming winter at the Albert Hall. One item on the list will occasion considerable surprise to those who have hitherto believed that the society confined itself almost entirely to oratorios and musical works which are not seen on the operatic stage. To show that the society moves with the times, one evening, it is announced, is to be devoted entirely to Wagner, and will include portions of the "Flying Dutchman" and "Lohengrin," in which Miss Esther Palliser and Mr. Andrew Black are announced to appear. The novelties comprise a choral and orchestral setting of Rudyard Kipling's "Ballad of the Camperdown," and of Coleridge Taylor's "Scenes from Hiawatha." Amongst old favourites we are promised "The Messiah," "St. Paul," and "Elijah," "The Golden Legend," and "The Redemption"; and Madame Albani, Miss Clara Butt, Miss Ella Russell, Mr. Ben Davies, and Mr. Santley, with other artists, are engaged to give their valuable services.

### Nursing in the London Poor Law School Infirmaries.

By a Past Matron.

### THE PROVISION FOR NURSES.

Before admission into a Poor Law school the children are obliged to undergo a medical examination, only those who are certified as healthy being passed. The others are drafted into the infirmary belonging to the union in London. Of course, this simplifies the nurses' work at the school infirmary very much.

The beds in the latter are usually classified under three headings—general sickness, ringworm and skin diseases, and ophthalmia—and each division is strictly isolated. One of the Local Government Board's rules directs that no child who is indisposed in any way is to be kept in the body of the school, but is to be sent up to the general sickness wards until seen by the doctor. The children's life being an exceedingly healthy one, it naturally follows that the greater number of their ailments are of a very slight description—headache in summer, and colds and chilblains in winter, form the bulk of the diseases that comes under the nurse's care, with occasionally a fractured limb caused by an accident in the playground.

From 1841 to 1874 ophthalmia in all its forms was the curse of the Poor Law school. Its cause was not known or, at least, generally recognised, and although many of the Boards of Guardians spent large sums of money in trying to stamp out the disease, it was not until Dr. Sidney Stephenson carried out his investigations for the Departmental Committee of the Local Government Board, and his report on the subject was officially published, that it was publicly understood to how great an extent the disease is preventable.

At the present time purulent ophthalmia is practically unknown in the Poor Law schools, and there are but comparatively few cases of either the muco-purulent or the chronic forms of the disease.

The great diminution of ophthalmia in these institutions is most interesting from a nurse's point of view, as showing what may be done towards the prevention of disease by care and cleanliness. In the old days certainly, attempts were made to keep the children clean, but alas ! the methods were lamentable. Instead of the modern separate bath in some of the schools, one large tank was provided in which many of the children could be bathed at the same time. Water was too precious to be changed very frequently, whilst flannels and towels were used promiscuously.

Care was not taken to separate any of the children who might have what were then known as "sore eyes" from the others. It does not need a very vivid imagination to picture the result of this casual treatment. If any of the various forms of ophthalmia was introduced into a school it soon became epidemic, and, indeed, in many instances it seems to have been in a fair way to become endemic at the time of Dr. Stephenson's investigations.

One of the chief factors in the prevention of ophthalmia has been the introduction of the shower-bath and jet spray system into the children's lavatories. The shower-baths are quite unlike those seen in any other institution. Imagine a row of porcelain niches, each with a porcelain pedestal, only needing a figure to complete its resemblance to some church screen or reredos. The canopy is lined with three or more water pipes, each ending in a brass bulb pierced with holes. The children sit on the little pedestals and wash in the water which is constantly flowing from the sprays over their heads. A gutter round the whole arrangement carries away the water, so that by no possible chance can a child wash or be washed in that which has already been used. A thermometer fixed into the supply pipe shows the heat of the water, and the attendant in charge can regulate its temperature by means of a special apparatus.

Along each side of the lavatory run long, low porcelain troughs, plentifully supplied with jet sprays acting on the same principle as the baths. Under no circumstances, therefore, can a child use any but running water direct from the pipes.

But all these elaborate and costly arrangements would have been of little or no use had the old plan of general towels and flannels been continued. At the schools where I was working, after many experiments, the Guardians decided to abolish flannels for washing purposes altogether. Every time a child entered the lavatory to wash, even if it were only its hands or face, it was allowed a clean towel half a yard long to use instead of the old-fashioned flannel, and as many more as were necessary of the same size for drying purposes. These towels were collected by the lavatory maid directly they had been used, and sent straight down to the laundry, where they were well boiled before being used again.

Next to ophthalmia, ringworm ranks as the bugbear of the Poor Law official. Fortunately, the measures already described have done much to stamp it out, but it is still far too common. The children are examined at their bathing (three times a week) by the matron or her deputy, but it is not by any means easy to always detect a ringworm in the early stage. If the doctor has already paid his daily visit it is a good plan to "paint" any doubtful spot; it will not do any harm, and may be the means of preventing an epidemic.

The nurse's quarters are, as a rule, comfortable. A superintendent or charge nurse has both a bed and sitting room, while a nurse is provided with a fair-sized bed-sitting room in one. The great drawback, of course, to rooms in any school holds good here ; it is impossible to get away from the children's noise when off duty ; for very few, if any, of the little patients suffering from ringworm or ophthalmia are confined to bed, and the nurse's rooms are so arranged that her sitting-room communicates with their day-room, and her bed-room with their night wards.

The rations for the nurse and her "helpers" (*i.e.* wardsmaids) are generally sent over from the main building every morning and cooked by one of the maids in the block kitchen. In this respect the nurse is more fortunate than the officials of the same rating in the schools, whose meals are cooked in the common kitchen, for whereas the latter are only allowed pudding or its substitute of pastry, &c., three times a week, the rations are so generous that the nurse will always find that, with a little management, she can contrive a decent dinner every day.

The times off duty are fairly good, and in summer the nurse can appreciate them, but winter tells another tale. The schools are generally in the country, and frequently some little way from a railway station. Unless the nurse is lucky enough to know someone in the village personally, she need never hope to make any outside acquaintance. The general public, including the clergy, as far as my personal experience goes, look askance at the Poor Law nurse, and would never dream of calling upon her, whatever her official or private position might be. I have already explained that off duty time indoors is useless to her on account of the children's noise. As for making friends indoors, well, at present the guardians seem to look upon domestic servants as the fitting persons to be in charge of the children, so I am afraid that it will be long before she finds congenial society among the officials in the schools themselves.

### Hospital Sketches.

### OLD TOM AND HIS WIFE.

Tom Raggett was admitted to the Royal Central West suffering from cancer. He had not come from Bloomsbury, or Marylebone, or Islington, or Clerkenwell, but from a small place in Bedfordshire, where hedge rows and hay-stacks are visible from the High Street. Tom's doctor, knowing that the necessary operation could not be performed at home without grave drawbacks, had persuaded the old fellow and his faithful spouse that the only chance of recovery lay in submitting to a journey to town and a few weeks' residence within the walls of a great London hospital. Tom was sixty-five and had been married to Jane in his twentieth year, and this their first parting for nearly half a century sorely tried them both.

"I woan't be away long, Jane," said Tom at parting. And Jane had only been able to reply, "No, Tom, doan't."

She and little Alice, their orphaned grandchild, who lived with them, had left the railway station and walked and sobbed the whole of the two miles that separated it from their little ochre-washed cottage. The old woman and the child of six met many friends as they trudged hand in hand back to the cottage without Tom, but none stopped and spoke to them, for all knew of their trouble. Tom was well-known to everyone in the place, and for miles around, and his ailment had been common knowledge for a long time.

As a youth and upwards till he was sixty-three Tom had worked as the village butcher's odd man. He had driven sheep and cattle and assisted in the slaughter house. In the way of outside work, when not wanted by the butcher, he had waited upon families whose pig had reached the desired weight, and who were anxious to have it killed beneath their noses and hoisted in pieces to the kitchen ceiling; he had obligingly poisoned dogs for neighbours, and put innumerable cats—with bricks—into bags for a specified purpose.

Curiously enough he was looked upon by everybody as the kindest-hearted man who ever lived, in spite of the fact that all went to him when they wanted murder done. After all it may have been that his success as a murderer was mainly due to his known soft heart, in precisely the same way that our cleverest surgeon is chaffed for his too tender-heartedness.

When Tom's health began to give way, two years previous to his visit to us, he had been obliged to forego the larger part of his weekly earnings, and he had in consequence been put in receipt of slender parish pay. He had said in confidence to a friend that he only wished he could have managed to live without the help granted him, for it had retarded his liberty in every possible direction. As he had accepted relief he could do nothing that he most wanted to do. He could not even wear a white waistcoat, and he had a "grand 'un" in his possession. It was a gift long ago from the wardrobe of a well-to-do patron. He had ventured to don it one fine Sunday last summer, and with simple pride he had gone out amongst his friends, but one of them had whispered the magically cruel word "pauper" in his hearing, and it had pained him terribly and made him so utterly wretched that he went home and took the waistcoat off and put it away in the darkness of an obscure nook. And it was "such a grand 'un," too! Poor old Tom Raggett.

Once in Chapman ward, and somewhat accustomed to his new surroundings, Tom found his tongue, and I had several conversations with him. One day he told me that he had been a great writer. His married daughter, who lived in Manchester, and his son Richard, who was a resident in New York, both expected letters from him regularly, and, although neither was in a position to remit the postal charges, Tom and his wife had "done fair" by them, and once a fortnight to Manchester and once a month to New York a letter had been duly despatched. Thus it was that Tom had been a great writer.

The day before Tom's operation, when he was on low diet and when he was naturally very weak and ill, I tried to cheer him by offering to write letters at his dictation to Manchester and New York, and, if he wished it, to Jane, his wife. He heard me with astonishment that gave way to rapturous pleasure, and the writing materials were produced forthwith.

"My Dear Daughter,—I told you in my last letter as how it were likely I would have to go up to London to be attended to in one of the big hospitals if I got no better. I come a week last Monday. It is a very big place is this hospital and as you knows well enough with your education it is stationed in a very big city. I do not know I can say I like it very much for all the kindness of the gentleman what is taking the trouble of writing off of my shoulders to you because there's a sight of things and faces and ways and noises as is mighty strange and queer to me as is fresh from the country as you knows well enough. My dear daughter I hopes as you are better than this letter leaves me, but please God I am only to be patient a bit more and then they will do something to me as will take away the pain I has in my inside and I will not be sorry either as it is dreadful and makes me I don't know what like. I hopes as you and your husband will never have it and that you will never know any of your friends either as will have it as it is as bad to bear till it makes me want to fall off of the bed and roll over and over. I am pleased to let you know that your mother is well at present and that if she did know as I was writing to you she would get me to say what I always does say from her to you. Her rheumatism was bad before when I wrote [Tom of course said 'writ'], but it went off very soon again and she has been right clear on it you will be pleased to know. When I gets back home again I will send you a long letter all about what they did to me at the hospital and I will tell you how I am then. It will not be a long job the doctor says and I hopes not either as this here pain is dreadful bad to have all the time as I am lying here away from your mother and little Alice and I miss them every hour and every time I looks round to see them when anybody comes this way near to where my bed is. I don't want to say over much to you this time as I am not writing with the pen myself for the kind gentleman as said he will write the letter to you and Richard and your mother for me. He says he will write them as my relations live over far off for them to come up here for to see me on the visiting days as is twice a week, and most of the visitors come here on a Sunday as that is the day when they don't have to go to their work and when they puts on their best things. Give my love to your husband and you accept the same for yourself from your loving father,

"Tom Raggett."

Tom just managed to scrawl his signature to the foregoing epistle, and he listened to me as I read it over to him with evident gratification. He showed surprise at the speed with which I was able to take down his words, and he told me that my method of writing was "a vast sight quicker nor" his own. A letter very similar to the one to Manchester was written to his son Richard. He would have preferred to give me the New York letter down word for word as he had done his daughter's, but his strength was not equal to it, and the Sister helped me to explain to him that the same letter would do for Manchester and New York if written out twice with small alterations and signed by him in each case.

"They won't exchange the letters, you know," weightily put in Sister.

The letter to Jane, his wife, was, however, far too important to be a mere copy of a letter to one of his children, and he insisted on dictating it himself syllable by syllable. "Let it be a *honest* letter," he said, with great emphasis. And I gathered from his half-sneer as he made the remark that he regarded me, notwithstanding my gratuitous services, as no better than the bulk of the low and crafty Londoners concerning whom he and his fellow-villagers held only a poor opinion.

"My Own Dear Jane,—It is very many years since I wrote to you as you knows as well as I do myself as there ain't been any call for it always having been together for nearly these last forty-six years. My dear wife how do you get on without your Tom? I reckon as how you misses me about the house and garden very nigh as much as what I misses you up here being as I am all amongst a lot of strangers though I don't say as some of them is not very good to me. But my dear Jane you know as I would a lot rather have you up here beside me to do for me and to get me the milk and other things as they lets me have than I would have these young women what wears white caps and long white aprons with scissors and chain and big pockets dangling about. I am going to be taken out of the ward to-morrow and carried to what they calls the theatre and then the doctor will take away what has been giving me all this here pain for these many days and then you will have me back again and I will be going about the same as what I have done all these years. When I come back I will bring Alice something nice as will be a surprise for her. I will get it at one of the fine shops as there is close to the hospital. And will you tell her I will do this my dear Jane and will you please give her a kiss for me and tell her that I will soon be home again with you and her now please God. The gentleman what is writing this for me has written to Ellen at Manchester and to Richard and he says that if I ain't well enough to leave the hospital in a few weeks that he will write for me to you all again. I want to come back to you Jane dear and I can't bear being away as I am not used to it and you ain't either and it makes it so hard for us when we have not been used to it. I can see you in the easy chair waiting for me to come for my tea and I can see little Alice untying my boots. My dear Jane I can't write any more. I want to shut my eyes and think about you and I want to see you and to feel your hand on mine. I will be home the first day as they will let me come to you. "Your loving husband, Tom."

The operation was successful enough, but the following day it was seen that Tom was not doing well. On Monday he was worse, and on Tuesday he was sinking. I therefore sent a telegram to his home doctor, asking him to kindly arrange for Mrs. Raggett's departure with all possible speed for the purpose of seeing her husband alive.

The old lady arrived, and was just in time to exchange a last, long kiss with Tom, and to hear him weakly murmur, "Doan't be long coming, Jane, dear."

### Presentations.

Coathill Hospital, Coatbridge.—A few days ago Nurse Elizabeth M. B. Hempseed was the recipient of a marble timepiece, given to her by the staff of the hospital, on the occasion of her marriage. Dr. Hamilton, visiting physician, made the presentation, and in the name of the staff wished Miss Hempseed every happiness in the life she was about to enter. A pleasant evening was afterwards enjoyed by the nurses. The timepiece bore the following inscription: "Presented to Nurse E. M. B. Hempseed, by the staff of Coathill Hospital, Coatbridge, on the occasion of her marriage, September 18th, 1899."

Budleigh Salterton Cottage Hospital.—A very handsome silver salver, together with a cheque for £45 3s. 1d., has recently been forwarded to Mrs. Warington, the late matron of this hospital. The salver bears the following inscription: "Presented with a purse to Mrs. George Warington, by the inhabitants of Budleigh Salterton and the district, in recognition of her voluntary services as matron of the Budleigh Salterton Cottage Hospital, 1888-1898." The names of the subscribers, about 180 in number, will be contained in a handsome illuminated album, the writing of the names and production of which has been undertaken by two ladies of Salterton. This will be forwarded to Mrs. Warington upon completion.

### The Matron's Corner.

### Rules to Make and Avoid.

"Make only few rules, but enforce those few absolutely," was the conclusion I arrived at in my last article. Make only few, for a multiplication of rules is apt to lead to pettiness, it is very much a woman's method of government. We women are not broad enough in our outlines, and breadth is an essential of good government.

Absolutely to enforce the rules you have formed is surely also essential, but nothing is more detrimental to the morals of the rules than the knowledge that it really does not matter whether rules are kept or not! There are certain standard rules which must of necessity be laid down in every hospital —rules of punctuality, of neatness, of accuracy, of order; these are essentials.

It may be argued that they are indeed too much matters of course to be insisted upon here. But unfortunately there are hospitals where even such every-day and necessary rules are allowed to lapse. The matron is pleasant, charming, kindly, but she does not enforce these elementary rules. There is a general lack of method in the wards—a haphazard way of doing everything. The rules exist, but no one enforces them. This is one of the cases in which the matron has let slip the reins of government. Everybody knows that she will not insist upon the keeping of her own rules—everybody breaks them. Yet, after all, it is so little trouble to enforce rules. True, it involves pulling people up every time a rule is broken, and this is disagreeable work for yourself; but when your subordinates once realise that your yea is yea, and your nay—nay, that if you say a thing must be done, well, it has to be done, you will be surprised to find in how short a time they fall into line.

Besides the enforcement of these elementary rules, ought not such rules to be enforced as those against standing about, and idling on stairs and in corridors, rushing about the wards (a thing I saw a sister and a nurse doing the other day in a big hospital!), professional conversation at meals, and an *outré* personal appearance. But, on the other hand, may I strongly urge that it is a grievous error to make a stringent rule as to the attendance of nurses at prayers in the hospital chapel. I do hold that it is right to have short prayers in each ward every day, at which the nurses of the ward should be present. But to insist that nurses should attend a chapel service morning and evening, whether they will or no, is worse than a mistake. Religion cannot be forced upon anybody, and, unfortunately, the more people are ordered to church, the less do they like going. I have heard nurses belonging to a certain hospital I know say, "They have given me a thorough sickening of church here. I've been obliged to go when I was tired and disinclined. When I leave hospital I shan't go at all!" For pity's sake, do not make rules about your nurses' religion.

Again, in one hospital I knew, nurses sleeping in the dormitories were forbidden to go into each other's cubicles. That rule could not be enforced, and was invariably broken; consequently it was a mistake. Though to make such a rule at all is a little bit of an absurdity, for why should grown-up women be treated like children?

May I end as I began—make few rules, enforce those few absolutely, and base your rules on big general principles of law and order, not on petty tyranny and childishness.

### To Nurses.

In order to increase and vary the interest in the *Mirror*, we invite contributions from any of our readers in the form of either an article, a paragraph, or information, and will pay a minimum of 5s. for each contribution. All rejected manuscripts are returned in due course, and all payments for manuscripts used are made at the beginning of each quarter, *i.e.*, January 1st, April 1st, July 1st, and October 1st.

### A Book and its Story.

### THE SCIENCE OF CHEIROSOPHY.

THE story told in the book that lies before us* is one of intense interest, whether or no the reader agrees with its tenets, for any subject having for its keynote the study of human character and destiny is sure to be read and appreciated by the large class to whom it appeals. Into the ethics of "Cheirosophy," proper or improper according to the view of the individual, we do not propose to enter, but to confine ourselves to the author's "argument" in its favour with which, in some well-written pages, he prefaces the opening chapters, and to the indications of character suggested by the form of the hands, as the book proceeds.

"The hands are the servants of the nervous system, and all things that affect the system affect them. . . . There are many people who would be amazed probably to know that in antiquity it is older than Christianity itself. Two thousand years before the birth of Christ it had its origin; and that away back in those regions where the snow-tipped Himalayas pierce the very heart of Heaven, this strange idea entered men's minds that the soul, somewhere and somehow, wrote out its own history—like the captive writing his name and legend upon the stones of his prison house, to be read or not, as the case may be."

The ancient Greek philosophers, "whose wisdom and knowledge has been the foundation of our modern schools of learning," found the science of the hand not beneath their notice. Even Anaxagoras, Aristotle, Paracelsus, Cardalius, Pliny, and Hispalius had great faith and respect for it, and we are told that the great Alexander himself received with favour a book on the subject forwarded by Hispalius to him, and in acknowledgment wrote: "It is a study worthy the attention of an elevated and inquiring mind." Later on, from receiving the attention of the scholars of the period, it drifted into the hands of charlatans and impostors, and in the early days of Church history we find an edict of ex-communication, and even of death, issued against those practising the art. But, wisely suppressed as it was—for it gathered around it the darker arts of witchcraft, black magic, and other undesirable developments—still the touch of truth was smouldering amid the shadows, and centuries later, in the year 1445, when the first addition of the Bible appeared with movable type, the Monk Hartlieb published a work on "The Study of the Hand," which, "rising Phœnix-like from the fires of persecution," fanned into life once more the ashes of the torch of inquiry.

In our own century science at last came to the rescue of "this so-called superstition." It began to be realised that "the hand is the immediate servant of the brain, under the direct influence of the mind and the still more mysterious influence and subtlety of thought. Meissner in '53 proved the existence in the hand of tactile corpuscles running in straight rows in the red lines of the palm." Everyone knows that the late Sir Charles Bell is one of the most distinguished authorities on the connection between the nerves of the brain and the hand. He considered that as the nerves from the brain to the hand are more numerous than to any other portion of the system, that as the action of the mind affects the whole body, it therefore follows that every thought of the brain more immediately affects the hand, and consequently its whole formation. "It has been proved lately that the grey matter of the brain can be found in the tips of the fingers of blind persons."

Whether the lines in the hand are due to the influence of the "subtle essence of the brain" or to that divinity "which shapes our ends roughhew them as we will," is perhaps of no great matter. The agency may for ever remain a mystery, but because at present it remains unsolved that constitutes no ground for its non-existence nor our unbelief. "As well," says Cheiro, "refuse to live because all that constitutes life is unknown to us or to refuse to think because the processes of thought remains unrevealed." From our own knowledge of Cheirosophy, and that neither a recent nor unvaried one, a popular fallacy is clearly disproved—that of the lines in the palms being caused by the folding of the hands. Nothing is more incorrect. Another very common error is that lines and furrows in the hand are the result of manual labour. You may find, and do generally discover in the hand of the student, the sedentary scholar, the society woman, or the invalid who never leaves the couch a network of clearly marked lines, wholly absent in the palms of those who are engaged in hard daily physical labour. Thought, nervous activity, too often nervous excitability, rays the hands, and when persons are physically inactive, it goes without saying they are generally more mentally awake, and this fine network of lines interlacing in the palm is the sign of mental alertness. To what good or mischievous use this will be applied depends on the type of hands in which the lines abound, and also as to their consistency, soft hands proving impressionability, sentimentality, indolence; hard hands the reverse—resistance, activity, and sensibility as opposed to sentimentality. The best consistency for a hand naturally is one between the two extremes, where resistance is tempered by impressionability, indolence by energy, and so on. Perhaps the most sceptical of our readers may like to have an opportunity of finding for themselves to which type their hands belong. Here are the seven received ones: First, the square, useful hand, "the hand that rules the world," with its finger having four distinct sides when held to the light. Lawyers, doctors, scientists, business men, succeed best whose hands are square. For they will then be tenacious, reliable, reserved, perhaps, but staunch in friendship and dependable in business matters. With spatulate-tipped fingers, the spirit of enterprise, discovery in mechanics and navigation, &c., with unconventionality, sometimes bordering on eccentricity, rule the possessor. Philosophic hands are long, have developed joints, and bony fingers. With this hand, independence of thought, especially in matters religious and political, courtesy in bearing to all, with familiarity to the few, are characteristics. The conic hand is one in which a love of luxury and indolence predominate. Quick enough in perception, the owners yet lack tenacity and patience to carry out their ideas. Lack of tact, in spite of a warmth of manner, makes them unconsciously brusque. They are ruled by emotion, and their apparent generosity is seldom disinterested; the weakness of the type is modified when the palm is firm and elastic; soft and cushiony, both ease and selfishness are indicated. The psychic hand belongs to the Idealists. The long, narrow hand, with slim, delicate, smooth fingers, shows the dreamer, the visionary, to whom the monotony and routine of life are a burden and a weariness. They are strongly affected by colour, and have been known to naturally succeed in art or design when in everything else they have failed." As a rule, it is given to the square or spatulate hands to work out the inspirations of this gifted but often hopeless type. The title of "mixed" is given to the last hand on the list. And the odd man or woman may easily apply the term as appropriate to their own hands when they learn that its characteristics belong to that large class of useful people who can turn their hand to anything; for, possessing many useful traits in general, and some gifts in particular, they are generally popular and useful members of a community. Cheiro's "Book of the Hand" is one of the best summaries of the subject we have seen, and it will afford amusement and profit for a dull half-hour, we feel sure, to those readers who, like the authority already quoted, possess "elevated and inquiring minds."

* "Guide to the Hand." By Cheiro. (London: Saxon and Co. 2s. 6d.)

### Broncho-Pneumonia in Children.

During the summer months the prevalence of broncho-pneumonia among children of all ages, and especially among those of the very poor is very noticeable. Children's hospitals and general hospitals are crowded with it (and with the more specially summer disease of diarrhœa); a women's ward in any large general hospital will often contain more children than adults owing to these causes. It is not, however, of the ordinary uncomplicated form of broncho-pneumonia that it is proposed to speak of in this short article, and for which ordinary care and nursing will suffice, and from which children seem to recover spontaneously, but of the rarer variety where the symptoms are much more severe, and the nursing more difficult.

Ricketty, delicate children who fall victims to broncho-pneumonia are liable to have it in a severe form. Head symptoms often show themselves early, perhaps soon after the fit that so commonly ushers in an illness in children; unconsciousness sets in with an occasional recurrence of fit or convulsion, and marked retraction of the head often appears, giving the child a clinical aspect of meningitis. There is high fever with no remissions for perhaps a fortnight or longer, great wasting takes place, and the prognosis and even diagnosis (from its similarity to tubercular meningitis with lung symptoms) are not easy to make at this stage. Feeding the child early becomes a difficulty, as all food is stoutly resisted, and, oddly enough, generally, if not always, by those children who ultimately recover, so that the fact of the child not taking nourishment willingly need not depress the attendants too much. As soon as it is evident that the child will not take food by mouth, it is better to avoid exhausting it by futile struggles, and resort early to nasal feeding, which, if skilfu[illegible] managed, is infinitely better for the child, and a more accurate way of giving nourishment.

For a child of three years old, ℥iii.-℥iv. of milk or beef-tea may be given every three hours; for a child of five years, ℥v. every three hours; but if there is much vomiting, lesser quantities must be given and oftener, though it is not desirable to pass the nasal tube too often. In many cases it will not be necessary to use the nasal tube for more than a day or two, while on the other hand it may have to be persevered with for a fortnight or more. If brandy is ordered, it may be given with the milk through the tube, but should the physician deem it advisable to give it oftener than every three hours, it is generally quite easy to get down five or ten drops in a teaspoonful of sweetened water every hour or so by mouth without much resistance from the child. Medicine is, of course, better given through the tube, and should be poured in separately to prevent any possible coagulation with the milk, which might block the tube. Vomiting is sometimes troublesome, and whey and albumen water can then be substituted for milk.

Diarrhœa often precedes and accompanies the fall of temperature and recovery, and as often the stools are passed involuntarily, constant care and watchfulness will be needed on the nurse's part, more especially when the child is much emaciated. It has been called the "critical diarrhœa," *i.e.*, the diarrhœa of the crisis, and as such is allowed to take its course by the physician. After the fall of the temperature, which takes place gradually with ascents and descents till it finally settles to normal, convalescence sets in and progresses rapidly. The desire for solid food and plenty of it is one of the first and most cheering signs of recovery, and the returning appetite is sometimes quite startling.

Occasionally when the attack has been protracted, and much wasting has taken place, convalescence is accompanied b[illegible] incessant crying, and a condition of "refusing to be comforted," which is very difficult to deal with, as all ordinary methods of consolation fail, and the child goes on wailing, to the distraction of its fellow patients, and the despair of the nurses. If food, warmth, and nursing after a good trial fail to relieve the child, it will be necessary to invoke the aid of the physician, as the child gets into a worn-out nervous condition, which the physical condition is unable to control, and of which it is probably the cause. Fortunately for all concerned, this irritable condition is not a common sequela of broncho-pneumonia, even in its severe form, and the majority get well with a surprising rapidity, considering the serious nature of the disease.

### Nursing the Hop-pickers.

By the Matron of the Little Hoppers' Hospital.

In reply to a request for an account of the nursing of the hop-pickers, when labours have just terminated, the Matron, Sister Dowie, sends us the following: "The Little Hoppers' Hospital, at Five Oak Green, a small village surrounded on all sides by hop gardens, and consisting of a four-roomed cottage, was opened last year for the benefit of the children of the hop-pickers, and it has been found to supply a real need. For the information of those who are ignorant of the hop-pickers and their ways, I may explain that during the months of the hopping seasons, roughly speaking, no less than 20,000 persons, mostly from the East End of London, are drafted into this part of the country. Entire families, down to the baby in arms (nay, even additions to the families are a constant occurrence during the season), come annually to the hop-farms for a 'holiday' and 'change of air.' When the weather is fine and warm the change of air may be very beneficial, but owing to the wretched accommodation provided for the pickers, wet, cold weather proves quite the reverse, especially for the children, who no matter how they ail, must either lie about in the fields exposed to the blazing sun or coldest wind, or else be left in the dark, draughty hop-house, tended only by another baby of a slightly large growth. To remedy this evil, the Rev. Richard Wilson, of St. Augustine's, Stepney, who comes down himself every year and works and lives amongst the hoppers, started this little hospital, which, since it opened its doors on September 2nd, has been filled with little patients, the majority of whom must have died had they been left where they were. Until now the work has been almost unknown, but people this season have most generously responded to Mr. Wilson's appeal, and have sent money to help on the good work, and clothes and toys which have delighted the hearts of the little ones and whiled away many a weary hour. A musical box which has been lent for their entertainment has proved a never-failing source of delight. The first request of a big girl of fourteen, and convalescent from pneumonia, on waking, constantly is: 'Please Nuss, make the music play,' and when the strains of 'Her Golden Hair' fade away, it is always, 'Oh! Nuss, make it do it again.' A delightful toy monkey has been the daily and nightly companion of a dear little mite, originally known as Mary the Contrary, but who, by the influence of that monkey, has been transformed into an angelic smiling baby with laughter and kisses for everyone. I think that when our Mary goes the monkey will have to go, too. Besides the in-patients there is a large out-patient *clientèle*, who come at any hour of the day and with every ache and pain imaginable. The duties of the nurses are not limited to tending the sick; they write letters for ladies who are unable to do so themselves, and welcome any gentlemen who, wishing to give drink a rest, is willing to step into the little front room and sign the pledge. It is a delightful work, and the nurses who are leaving it will do so with many regrets. More money is needed to carry on the work next year. This will doubtless be forthcoming when the hospital reopens next season, which we hope may prove as successful a one as this has been."

### Everybody's Opinion.

[Correspondence on all subjects is invited, but we cannot in any way be responsible for the opinions expressed by our correspondents. No communication can be entertained if the name and address of the correspondent is not given, as a guarantee of good faith but not necessarily for publication, or unless one side of the paper only is written on.]

### STREET COLLECTIONS BY PERSONS IN NURSES' DRESS.

"Holiday" writes: We hear a great deal about the degradation of nursing uniform by the flighty and obtrusive nurse and others who masquerade in the dress to attract notice in the public thoroughfares. These two evils must be endured, as they cannot be cured. But surely something can be done to stop the street collections by girls in caps and aprons, such as took place at the Isle of Wight last week in aid of the Isle of Wight Infirmary. If the people who patronise confetti fights wish to help the infirmary, surely it is not necessary for the nursing staff to be present to receive donations. A few automatic machines would pay as well if placed in or near the field of action, and both nurses and visitors would be spared much unpleasantness.

### "IS THE NURSING PROFESSION OVERCROWDED?"

"An Old Sister of the Hollond Institution," commenting on the note in the *Gentlewoman* to which we alluded last week, writes: With the "hundreds of nurses in London who have nothing to do" I have nothing in common, but I should just like to say that a nurse who has conscientiously gone through her period of training, who wishes to succeed, and who is a nurse with true nursing qualifications, need never join the "hundreds in London who have nothing to do." With regard to nursing abroad, the Hollond Institution (which I know receives honourable mention in your paper, and for which I have worked some years, and hope to work many more) was founded in 1878 by the late Mrs. Robert Hollond, who had just experienced much trouble and anxiety through being unable to obtain an efficient trained nurse abroad and was desirous of saving others from suffering in a similar way. Since her death (1884) it has been continued by her cousin, Mrs. Woodrooffe. Upwards of one hundred nurses thoroughly trained and certificated are now employed by this institution, homes attached to which are established in Paris, Nice, Cannes, and Hyères. The nurses are appreciated by all the doctors (French, German, English, American, Italian) for whom they work, and they are generally in requisition.

### NURSING IN IRISH WORKHOUSES.

"An Irish Matron" writes: Many of us consider that the recent order issued by the Irish Local Government Board regarding trained nurses in the workhouses, though a step in the right direction, was done without due consideration for the provincial hospitals and infirmaries. It would seem more just to have had a term of three years' training laid down, and then some recognised standard or examination to go by. As things are now, in many unions the order will remain a dead letter. The Dublin nursing schools will be expected to chiefly supply the demand. So far, they have proved inadequate. Capable nurses willing to take the positions are not forthcoming. I think there are several hospitals in Ireland where great care is taken to train probationers, and which can turn out quite as good a nurse as any of the large hospitals. I acknowledge that we have one disadvantage to contend with, namely, hospital discipline and strict punctuality in the details of our daily work. This is almost beyond the control of the nursing staff. In the clinical schools the time of the surgeon's visit is known; he always comes at a certain time to meet the class; there is a programme, as it were, of the morning's work, so you are ready for each event, and no time is wasted. In the smaller institutions the doctor often has other appointments and work which may be quite as important to attend to, so he comes at the most convenient hour. When three or four doctors visit at uncertain times, either together or separately, people connected with hospital management will understand that the wheels cannot run quite so smoothly, nor can the morning's work be mapped out so accurately as it otherwise might. This, I admit, is a drawback; but the nurse who is obliged to accommodate herself to her surroundings may not necessarily be the worse as regards her knowledge and capabilities.

### BREAKFAST IN THE BEDROOM.

"Morganwg" writes: I beg to say a few words in reference to "Bee's" remarks about "Breakfast in the Bedroom." I am also a private nurse of fourteen years' standing. During that period I have received kindness and consideration from most of the families I nursed in. One or two have been most thoughtless, apparently supposing that the poor nurse never requires rest and ought never to be tired. The latter are the kind of people who seldom give their nurse a thought as soon as the case is at an end, in spite of the unwearying attention and pains the latter may have taken with her patient. On the other hand, there are many who will never forget all their nurse has done for them. As to breakfast in the bedroom, I have never been asked to have mine either in my patient's or my own bedroom; but I always take it in the patient's room on principle, one reason being that in many cases there has been no one to leave with the invalid. Again, I so often find that the family breakfast hour does not suit me; it interferes with my work, as I am generally busy at that hour getting my patient, &c., ready for the doctor. Where there is a family or friends in the house I think it only right that one of them should offer to relieve the nurse for a while each day, when the patient is well enough to be left to them, but even then I do not care about it, as so often I have found on returning that my patient is not so well. As to meals in the kitchen, no family that I have nursed for has ever suggested sending me to the kitchen. I think in many cases where a nurse has had her meals with the servants she has herself chiefly to blame for it, as she should have refused at the outset to take them in the kitchen, and gradually have shown the family by her manner that she was not accustomed to associate with servants. Still, I have known nurses who, although they have been shown every attention by the family, have not been happy unless mixing and gossiping with the maids in the kitchen. My opinion is that a nurse will gain respect both with the family and with the servants, too, if she upholds her position and is kind and courteous to all.

"Incredulous" writes: May I be allowed to say a few words to "Edith's" opinion, not only of "Breakfast in the Bedroom," but also "As to how a nurse is treated generally." I have had considerable experience in private nursing both in England and the East, and I have always, both by the well-to-do and those not so well endowed with this world's good things, received the utmost kindness and consideration from members and friends of my patients' home and family. I am sorry for "Edith's" lot; hers has evidently been an unique experience to say the least of it, and one wonders her training in hygiene did not appeal to her in suggesting more healthy arrangements respecting meals? I did not before know that "bounce and assurance" were recommendations to a nurse in a doctor's opinion, and I fancy I should fight shy of such a man did I ever hear of him. My experience has taught me that quietness and order in speech and manner, attention to little things and strict adherence to doctors' rules have not only been recommendations in my career, but absolutely necessary to successful nursing. Bounce and assurance in a sick room! or even in a well ordered household! Small wonder people dread "a trained nurse." A nurse's duties are to attend to the sick and suffering at the call of duty. Fancy our "soldier nurses" inquiring into the character, &c., of a "case" before she ministers to a sufferer's wants! Why should a nurse think it necessary to "stand on her dignity" and torture the brains of an anxious relative or friend for the "character and moral standing" of a patient and their surroundings before going to "the call of duty"? If a nurse is a lady, she will behave as such in every detail, and consequently receive in return not only respect from servants, but also courtesy, confidence, consideration, and every kindness from each inmate of a troubled and worried house to which she may be called, sometimes too hurriedly to even think of "references" and "all particulars." One must also guard against enthusiasm for "flowers" and "Sunday School teaching," tempting one to forget there is a "sick-room and its duties."

### For Reading to the Sick.

ST. MICHAEL'S DAY.

Are they not all ministering spirits, sent forth to do service for the sake of them that shall inherit salvation.—*Heb.* i. 14.

He shall give His angels charge over thee, to keep thee in all thy ways.—*Psalm* xci. 11.

**Reading.**

It is I confess my great sin, that I have filled mine eyes with other objects, and have been slack in returning praises to my God, for the continual assistance of those blessed and beneficent spirits, which have ever graciously attended me, without intermission, from the first hour of my conception to this present moment; neither shall ever (I hope) absent themselves from my tutelage, and protection, till they shall have presented my poor soul to her final glory: Oh, that the dust and clay were so washed out of my eyes, that I might behold, together with the presence, the numbers, the beauties and excellencies of those my ever-present guardians. What shall we then think of Thee the great King of eternal glory, that hast before Thy throne innumerable hosts of powerful and glorious spirits of Thine own making and upholding? And how safe are we under so many and so mighty protectors? It might be perhaps well meant, and is confessed to be seconded with much reverend antiquity, the conceit, that each man hath a special angel designed for his custody; and if but so, we are secure enough from all the danger of whatsoever hostile machinations. The beloved Disciple in Patmos, as by inspiration from that God, says, "I beheld, and I heard the voice of many Angels round about the throne, and the Beasts, and the Elders, and the number of them was ten thousand times ten thousand, and thousands of thousands"; and if so many were about His throne, how many do we think were about His missions? O ye blessed spirits, ye are ever by me, ever with me, ever about me; I do as good as see you, for I know you to be here; I reverence your glorious persons, I bless God for you; I walk awfully because I am ever in your eyes, I walk confidently because I am ever in your hands.—*Bishop Hall.*

Angels thy old friends there shall greet thee,
Glad at their own home now to meet thee,
All thy good works which went before,
And waited for thee at the door,
Shall own thee there: and all in one
Weave a constellation
Of crowns, with which the King, thy spouse,
Shall build up thy triumphant brows.

All thy old woes shall now smile on thee,
And thy pains set bright upon thee:
All thy sorrows here shall shine,
And thy suff'rings be divine.
Tears shall take comfort and turn gems,
And wrongs repent to diadems.

And so
Thou with the Lamb thy Lord shalt go,
And wheresoe'er He sets His white
Steps, walk with Him those ways of light,
Which who in death would live to see,
Must learn in life to die like thee.

—*Richard Crashaw to S. Teresa.*

To weary hearts, to mourning homes,
God's meekest Angel gently comes:
No power has he to banish pain,
Or give us back our lost again;
And yet in tenderest love our dear
And heavenly Father sends him here.

—*J. G. Whittier.*

### Notes and Queries.

**The contents of the Editor's Letter-box have now reached such unwieldy proportions that it has become necessary to establish a hard and fast rule regarding Answers to Correspondents. In future, all questions requiring replies will continue to be answered in this column without any fee. If an answer is required by letter, a fee of half-a-crown must be enclosed with the note containing the enquiry. We are always pleased to help our numerous correspondents to the fullest extent, and we can trust them to sympathise in the overwhelming amount of writing which makes the new rules a necessity.**

**Every communication must be accompanied by the writer's name and address, otherwise it will receive no attention.**

*Permanent Home.*

(230) Could you inform me of any permanent invalid homes in a bracing sea air for ladies of small means? I am a governess with a small income, and am suffering from a nervous complaint. I am anxious to hear of some home where I could be received if my complaint becomes too pronounced for the boarding-house where I am now living, and where I could have nursing if required.—*E. M. S.*

Some of the advertisements in The Hospital might suit you; and it would probably be worth your while to advertise.

*Crippled and Incurable.*

(231) I should be much obliged if you could tell me if there is any institution where a crippled lad of eighteen would be taken free. He is active, but has incipient phthisis. His is a peculiarly sad story, and at present his only home is the workhouse.—*Matron.*

The Thompson Memorial Institution for Incurables, Lisburn, seems the only one suitable to the case.

*Typhoid Convalescents.*

(232) Can you tell me of a convalescent home near Rugby where a man of forty-one and a boy of fourteen could be sent later? Both are recovering from typhoid fever.—*District Nurse.*

So many circumstances, respecting both patient and home, of which we know nothing, make it impossible for us to select a home for you. Why not get a copy of Burdett's "Hospitals and Charities?" The price is only 5s., and it contains full and reliable information upon all accredited institutions. It is published by the Scientific Press, W. The nearest home to Rugby appears to be the Wingfield Convalescent Home, Headington, near Oxford. It is for men, women, and children, and the payment is 7s. a week each.

*Q.V.J.N.*

(233) Will any Q.V.J.N. kindly give some particulars of this work, hours off duty, hours on, and holiday time? In fact, any information concerning the Q.V.J.N. to a trained certificated nurse desirous of joining.—*W. H.*

There is no uniform code of rules and regulations for Queen's nurses. Each local committee, with the approval of the institute, frames its own, in accordance with the requirements of the case. Apply to the General Superintendent, Queen Victoria's Jubilee Institute for Nurses, Queen Katherine's Hospital, Regent's Park, N.W., for further particulars.

*Stewardess.*

(234) "E. P." writes that about three weeks ago she applied to two of the largest mail boat offices for the position of stewardess. One line only employed experienced stewardesses, and the other had no vacancies nor was likely to have them.

We cannot do better than refer our disappointed correspondent for consolation to "A. C.'s" letter on "Nurses as Stewardesses" in the "Mirror" for the 16th inst.

*Dandruff.*

(235) Will you kindly tell me how to remove dandruff from the scalp, and to prevent the hair from falling out? I have used borax and a fine comb, but without effect.—*A.*

Dandruff generally indicates something wrong with the general health of which the scalp trouble is only a symptom. A useful article on the local treatment appeared in The Hospital for July 31st, 1897. It is by Leslie Phillips, M.D. Wear a warm nightcap and sleep with the windows open.

*Probationers for Military Hospitals.*

(236) Will you kindly let me know if they take probationers to train in military hospitals? If so, to whom do I apply?—*Inquirer.*

Probationers are not trained in military hospitals, but fully-trained lady nurses accepted for the Army Nursing Service receive a course of final training at Netley.

*Diabetic Patients.*

(237) Can you, or any of your readers, give me the address of the firm that caters for diabetics?—*Nurse Clara.*

There are several. Messrs. Callard, Regent Street; the "Manhu" Diabetic Food Company, 88, Charing Cross Road; and Messrs. G. Van Abbott and Sons, 104, Wigmore Street, Cavendish Square, are well-known advertisers.

*Allopathic Treatment.*

(238) Edith D. wants to know the name of a good book on the allopathic treatment of illness. The same sort of book that Dr. Ruddock and Dr. Fleming have written on homœopathic treatment. The book is for a children's nurse, who cannot afford more than 5s.

"A Handbook for Nurses," by J. K. Watson, M.D. (Scientific Press, price 5s.), gives a great deal of information about all common diseases. But the medicinal treatment of disease is hardly a subject for a child's nurse to take up.

### Travel Notes.

By Our Travelling Correspondent.

### XXXVI.—HEIDELBERG.

You may take a holiday at Heidelberg very reasonably, and there is no place that you would like better if you care for beautiful wooded walks, which abound above the Schloss and on the banks of the Neckar. In a fortnight, which is as long a holiday as many nurses can afford, you will have thoroughly explored the country round Heidelberg and laid up a store of pleasant remembrances on which to meditate in the night watches or in the wearisome time spent when your patients are perhaps convalescing from fever, and there is hardly anything to do for them, a state of things when time often hangs heavily with an energetic nurse.

### The Journey and its Cost.

The cheapest way, and a very agreeable one, is by the Netherlands Steamboat Company from Blackwall or the Tower. Second-class return only £1 11s. 7d. to Mannheim, from which place Heidelberg is distant five miles. There is but one drawback to this agreeable mode of travelling, and that is the time it occupies. The entire journey from London to Mannheim by steamer takes four days and nights. Thus, reckoning both ways, the time spent in the actual journey would be eight days—too long a time to spare on a short holiday. But if you can be sure of three weeks' rest, the steamer route enables you to see all the beauties of the Rhine—it rests you body and mind, and last, and by no means least, it is ideally cheap. Between £1 11s. 7d. by water and £3 12s. 4d. by rail there is a vast and most pleasing difference.

### Accommodation in Heidelberg.

I prefer the Schloss Hotel above the castle to all other resting-places. Terms from six to ten marks. You must always remember that a mark is the same as a shilling. If you wish to economise I think you would do well to write beforehand and try for rooms at the Pension called Lang's Private Hotel—pension, four to seven marks—or the Beau Séjour Anlage 32, or the Nebel Karl Strasse 16. Terms at all these start from four to five marks.

### Attractions in the Town.

First and foremost naturally comes the Schloss. Its splendid position is familiar to most from pictures and photographs, though none of these can adequately set forth its charms, the little town occupying for the most part one long street by the side of the Neckar, which is crossed by a fine old bridge, nestles snugly under the closely-wooded heights of the Jütta Buhl on which the Schloss stands. There are three ways of ascending, firstly, prosaic, but comfortable, the wire rope railway close to the Prince Karl Hotel; secondly, what is called the New Road; and thirdly, far preferable to the others, the Burgweg, which leads in fifteen minutes or so to the balcony and courtyard. By this pathway the castle was surprised and taken many a year ago, but in what particular war I have forgotten. The history of its many sieges is most confusing, and the most well-arranged brains find it far from easy to disentangle the mass of historical incidents which surround the noble pile.

### The Schloss Itself.

It was founded by the Count Palatine Rudolph I. in 1294. Rupert III. added to it in 1410; it was further fortified at the end of the fifteenth and beginning of the sixteenth centuries; Otto Heinrich and Frederick, husband of Elizabeth, daughter of James I., added the portions which go by their names. It was in the reign of Louis XIV. of France that Heidelberg suffered the most severely, and in 1764 fire caused by lightning reduced it to its present condition. I must not stop to tell you much of its beauties now, and I hope you will see them for yourselves. The Great Balcony is my favourite spot, and here and through the stately courtyard you may wander at will, sketch, photograph, or be utterly lazy, no one will interfere with you. You will be very lucky if you can see it once under a brilliant moon.

### Trinkhalle Behind the Schloss.

Several times a week a most seductive band discourses sweet music, whilst you sip your coffee in the open-air restaurant in the Schloss Gardens; and what melody it is, a revelation, indeed, after the appalling German music of the streets, under which we rage and swear in London.

### Excursions.

You will be sure to ascend to the Molkencur, walking twenty minutes, wire rail three minutes. From there you can go on to the Königstuhl; it is three-quarters of an hour's walk, but through lovely shaded woods. By crossing the Neckar, gaining the opposite bank, and turning to the right, you will find innumerable lovely walks up the valley in all directions. A steam tramway goes to Weinham, and a walk of two miles takes you to Philosophenweg. In term time it is interesting to see the students in their different gay caps parading the streets. There is a still a great deal of duelling, which results in much mutilation, sometimes of the faces and fingers of the warriors, but does not end fatally. The only church of any interest is the Heilig-Geistkische, which is fifteenth century; round its feet surges the busy life of market days; immediately opposite is the Zum Ritter Inn, of the same period and in excellent preservation. The Rathhaus is comparatively modern, 1701. The repeated sieges, burnings, and internecine struggles have left but few specimens of early domestic architecture, and even the university buildings do not date further back than early in the eighteenth century. It is worth while to devote one day to taking the railway journey up the valley of the Neckar from Heidelberg to Neckarelz and back, it gives a good general idea of the richly-wooded country and luxuriant vineyards.

---

### TRAVEL NOTES AND QUERIES.

Switzerland (Shy).—There are several agencies that supply cheap tickets and accommodation in Switzerland, and you will do well to employ one. You do not tell me how long you wish to spend abroad, nor how much money you can spare, therefore my help can only be vague. Dr. Lunn has a tour of Paris, Geneva, Chamonix, Zermatt, Rhone, Glacier-Furka Pass, Andermatt, St. Gothard Railway, Lucerne, and Berne for 22 days. Price £15 10s.; this will include hotel accommodation for 13 days. The other days must be paid for separately. A much cheaper tour is one for £6 6s. to Geneva, with seven days hotel accommodation. The ticket enables you to remain longer, and you can make excursions in the neighbourhood. These particulars will enable you to think the matter over. Write to me again next year, telling me how long you mean to stay and how much money you wish to spend, and I will give you further information.

Ober Ammergau (Stevenage).—Look in this column about the end of October. I shall publish some arrangements for the Passion Play as soon as the railways have made their plans. It will be necessary to book places early.

---

### Death in Our Ranks.

On September 21st there passed away, at the Birmingham General Hospital, after a very short illness, Nurse Catherine Gordon, aged 26, who was in the second year of her training, having entered as a probationer on December 7th, 1897. The interment took place at Witton Cemetery on the 25th inst., the funeral ceremony being performed by the Rev. Father O'Dowd. On the coffin were crosses and wreaths from Miss Gordon (sister), the Rev. S. G. and Mrs. Collier, Miss Marriott, Mr. and Mrs. Barling, Sisters Coghlan and DeWind, a former patient, and from the matron and nursing staff, the residents, the house governor, and the servants of the General Hospital.

